# Treatment outcomes and adherence to treatment in patients with immune thrombocytopenia in two Ethiopian teaching hospitals: a retrospective cohort study

**DOI:** 10.1038/s41598-024-62372-w

**Published:** 2024-05-24

**Authors:** Dessale Abate Beyene, Eskinder Ayalew Sisay, Atalay Mulu Fentie, Amha Gebremedhin

**Affiliations:** 1 Department of Pharmacology and Clinical Pharmacy, School of Pharmacy, College of Health Sciences, Tikur Anbessa Specialized Hospital, Addis Ababa University, Addis Ababa, Ethiopia; 2 School of Medicine, College of Health Sciences, Tikur Anbessa Specialized Hospital, Addis Ababa University, Addis Ababa, Ethiopia

**Keywords:** Immune thrombocytopenia, Treatment outcomes, Platelet count, Corticosteroids, Ethiopia, Medical research, Outcomes research, Haematological diseases, Immunological disorders

## Abstract

The treatment of immune thrombocytopenia (ITP) is challenging and treatment outcomes depend on numerous unknown and patient-specific factors. Corticosteroids are the cornerstone of ITP treatment, but they are associated with many side effects. In this retrospective cohort study, treatment outcomes and treatment adherence in patients with ITP were investigated in 214 ITP patients from November 15, 2022 to March 15, 2023. Multinomial regression analysis models were used to identify predictive factors for treatment outcomes. A *p* value of less than 0.05 was considered statistically significant. Most study participants were female 161 (75.5%), and the majority 172 (80.4%) of them were taking prednisolone only. In terms of treatment adherence, 178 (83.2%) of the study participants adhered well to their ITP medications. The complete response rate at 3 months was 139 (65.0%). Predictive factors for partial response were increased negative impact of ITP on health-related quality of life (AOR = 1.221, 95% CI 1.096–1.360), being treated at Tikur Abessa Sepcialazed Hospital (AOR = 0.431, 95% CI 0.197–0.941) and the presence of heavy menstrual bleeding (AOR = 2.255, 95% CI 0.925–5.497) compared to patients with complete response. Hepatitis B virus-infected ITP patients (AOR = 0.052, 95% CI 0.004–0.621) were also a predictive factor for no response compared to complete response.

## Introduction

Immune thrombocytopenia (ITP) is an acquired form of thrombocytopenia and bleeding disorder caused by autoantibody-mediated and cell-mediated destruction of platelets, resulting in accelerated platelet clearance and impaired thrombopoiesis^1,2^. It is described as a transient or persistent reduction in platelet count < 100 × 10^9^/L and an increased risk of bleeding that depends on the degree of thrombocytopenia^2,3^. Autoantibodies are considered the main cause of thrombocytopenia, and binding of antiplatelet autoantibodies to their antigenic glycoprotein leads to elimination/phagocytosis of platelets in the reticuloendothelial system by monocytes/macrophages, primarily in the spleen^4,5^.

The incidence of ITP varies and is most common in young adults, particularly women of childbearing age, with a female-to-male ratio of 2 to 1 and an estimated 1.6–4.4 per 100,000 person-years^6–8^. According to the guidelines of the American Society of Hematology (ASH) of 2019, ITP is classified into primary and secondary forms depending on the cause^9^. In the adult population, more than 20% of ITP patients have a secondary etiology with infections, autoimmune diseases, malignancies, and certain medications, such as heparin, carbamazepine, linezolid, rifampicin, and vancomycin^10–14^. Coronavirus disease (COVID-19) has also caused moderate ITP after one week of presentation and diagnosis^15^.

The presentation of ITP varies among patients and some experience life-threatening bleeds, while others have mild bleeding with mild mucocutanaeous or subcutaneous haemorrhages^16,17^. Signs and symptoms typically occur with platelet counts below 100 × 10^9^/L, but bleeding is unlikely until platelet counts drop below 30 × 10^9^/L^18^. The patient presented with epistaxis, mucosal bleeding, skin manifestations (petechiae and ecchymosis), and visceral bleeding (gastrointestinal, cerebral, and gynecological bleeding) and fatigue during adimision^17,19–21^. Fatigue is a common symptom of ITP, affecting 22% up to 58% of patients and was found to remain prevalent and debilitating throughout the course of the disease^17,22,23^. In addition to fatigue, patients with ITP may also have other symptoms such as anxiety, headaches, depression and weight loss^23,24^. ITP treatment is challenging, and treatment outcomes are assessed based on the clinical response rate, treatment relapse rate, platelet count before and after the treatment, and adverse events of the treatment/procedure^2,9,25–29^. Therefore, treatment should be tailored to the individual patient, considering factors such as age, lifestyle, comorbidities, compliance, patient preferences, the presence and severity of bleeding, and the potential treatment side effects^30^.

Currently, there are three main approaches in ITP treatment, namely, suppression or modification of abnormal immune responses (using corticosteroids or rituximab), suppression of platelet clearance (using splenectomy), and stimulation of platelet production [using thrombopoietin receptor agonists (TPO-RAs)]^31^. Corticosteroids are the standard first-line treatment for adults with ITP who require treatment, and there is no relative contradiction with the addition of intravenous immunoglobulin (IVIg) in patients with active bleeding or those who prefer a rapid increase in platelet count^32^. Due to the unavailability of IVIg and TPO-RAs, clinicians in Ethiopia mainly used corticosteroids as first-line treatment and rituximab, azathioprine, and splenectomy as second-line treatment. Immune thrombocytopenia is persistent or chronic in approximately 70% of adult cases, and a long-term response is observed only in 25% of patients. Approximately 60–70% of adult patients require additional treatment either because they cannot tolerate steroids or because of relapse, and 98% of ITP patients taking steroids reported at least one adverse event^2,26,33,34^. Immunosuppressive therapy, especially high-dose corticosteroids and prolonged use, predisposes patients to infections and is significantly associated with poor treatment outcomes^35^. To reduce corticosteroid toxicity or complications, ASH Guidelines and an international consensus report recommend that corticosteroid use should be limited to a short period^2,9^.

To our knowledge, there is still no published evidence-based literature performed in Ethiopia or Africa that assesses the treatment outcomes, adherence to treatment, and corticosteroid common side effects. Hence, this study aimed to investigate the treatment outcomes and to determine the factors associated with the treatment outcomes of ITP patients in Tikur Anbessa Specialized Hospital (TASH) and St. Paul's Hospital Millennium Medical College (SPHMMC), the two teaching hospitals located in Addis Ababa, Ethiopia.

## Materials and methods

### Study setting

This study was conducted at TASH and SPHMMC, where specialized comprehensive and clinical services are provided. The outpatient departments of both hospitals serve patient at their differnt specialty clinics, and the hematology clinic is the biggest one. According to the hospital's Health Management Information System (HMIS) data of TASH and SPHMMC, an average of 50 ITP and 20 ITP patients visit the hematology clinic per month, respectively.

### Study design and period

A retrospective, hospital-based cohort study was conducted to assess treatment response in ITP patients who were on follow-up in the hematology clinic of TASH and SPHMMC during the study period. Data on adherence to treatment and corticosteroid side effects were collected directly from patients from November 15/2022 to March 15/2023.

### Eligibility criteria

Eligible patients included were (i) all patients attending both hospitals during the study period who had a confirmed diagnosis of ITP according to the guidelines of ASH of 2019 and the standardization of terminology, definitions, and outcome criteria in ITP of adults and children (primary, secondary, newly diagnosed, persistent, chronic and severe ITP)^2,32^. (ii) Patients who had been taking treatment for at least 3 months. (iii) Patients aged ≥ 14 years and (iv) patients willing to participate. Patients who did not start treatment or had received treatment for < 3 months and incomplete medical records were excluded.

### Sample size determination and sampling technique

Due to the rarity of the incident, all ITP patients that fulfilled the eligibility criteria who visited TASH and SPHMMC during the study period (4 months) were recruited. . Study participants were recruited from TASH and SPHMMC using a consecutive sampling technique.

### Data collection and management

#### Data collection instruments

##### Data abstraction form

The data abstraction format is designed to extract information from the medical record and directly from the patient, such as sociodemographic characteristics (age, sex, educational status, and place of residence), clinical and pathologic characteristics (type of ITP, duration of symptoms, comorbidity, laboratory, and clinical findings at diagnosis, presence of bleeding, platelet count at baseline and during data collection, and phase of ITP), treatment-related characteristics (type, frequency, and duration of treatment), and follow-up related characteristics (disease recurrence, in-hospital medical events, surgical complications, treatment relapse, and refractoriness).

##### Treatment outcome tools

After an extensive review of the literature^36–39^ and with the help of experts, structured questionnaires were designed to evaluate the treatment outcomes of ITP patients.

##### Morisky Green Levine scale (MGL)

The MGL is in the public domain and is widely cited in peer-reviewed journals. It was originally developed and validated for patients with hypertension to assess self-reported medication-taking behaviour^40^. Later, the scale was used to assess medication adherence in patients with various chronic conditions^41,42^. The scale contains four items with a score of *“Yes”* = *1 and “No”* = *0*. The items are summed to obtain a score range of 0 to 4. The scores were rated as follows: good adherence (MGL = 0) and poor adherence (MGL ≥ 1).

### Data quality assurance

A pretest was administered to 5% of ITP patients. The purpose of the pretest was to ensure that respondents understand the questions and can review the wording, logic, and skip order in a way that makes sense to respondents. Based on the results of the pretest, appropriate corrections were made on the data collection tool before the actual study was conducted. Data collectors were recruited by two clinical pharmacists and one nurse, and a half-day training was given by the principal investigator about the objectives of the study and how to use the tool to collect data directly from the patient and medical records/charts. To ensure quality, completeness and consistency of the data, all data were reviewed daily by the principal investigator.

### Data analysis

Data were entered and analyzed using Statistical Package for the Social Sciences (IBM Corporation, Armonk, NY, USA) version 26. Descriptive statistics such as frequency, median, and inter quartile range (IQR) were used to summarize the sociodemographic data and clinical and treatment characteristics. After checking the assumptions, univariate analysis was performed to obtain candidate variables for the multivariable regression model to determine possible predictors of the treatment outcome variables. In the univariate analysis, factors associated with treatment outcome that showed a marginal association at *p* < 0.2 after univariate analysis and all clinically relevant variables were considered candidate variables for the multinomial regression model to identify strong factors associated with treatment outcomes. A *p* value < 0.05 was considered to indicate statistical significance.

### Ethical consideration

Ethical approval for the study and study protocol was obtained from Addis Ababa University, College of health science, School of Pharmacy ethical review board (approval number: ERB/SOP/487/14/2022). The aims of the study were clearly explained to the study participants. The information was collected after obtaining written informed consent from each participant and taken from participants’ family/legal guardian for participants whose age was between 14 and 18 years. The right was given to the study participants to refuse or discontinue participation at any time they wanted and the chance to ask anything about the study. For obscurity, the participant’s name was not used at the time of data collection, all other personnel information was kept entirely obscure, and confidentiality was assured throughout the study period. Moreover, all methods in the present study were performed in accordance with the declarations of Helsinki.

### Operational definition

*Complete response (CR)* A platelet count after treatment ≥ 100 × 10^9^/L measured on two occasions > 7 days apart and absence of clinically relevant bleeding.

*Partial response (PR)* Platelet count ≥ 30 × 10^9^/L and a greater than twofold increase in platelet count from baseline measured on 2 occasions > 7 days apart and the absence of bleeding.

*No response (NR)* Platelet count < 30 × 10^9^/L or a less than twofold increase in platelet count from baseline measured on 2 occasions > 7 days apart or the presence of bleeding.

*Newly diagnosed ITP* Within 3 months from diagnosis.

*Persistent ITP* Between 3 and 12 months from diagnosis.

*Chronic ITP* Lasting for more than 12 months from diagnosis.

*Corticosteroid-dependent ITP* The need for ongoing or repeated doses of corticosteroids for at least 2 months to maintain a platelet count at or above 30 × 10^9^/L and/or to avoid bleeding (patients with corticosteroid dependence are considered nonresponses).

*Adherence* The extent to which a person’s behavior corresponds with recommendations from health care providers.

- *Good adherence* was determined when those study participants' Morisky Green Levine scale scored = 0.
- *Poor adherence* was determined when those study participants on the Morisky Green Levine scales scored ≥ 1.

*HRQoL* is a measure of how much ITP has affected the patient’s life in recent month in terms of ability to perform daily activities, maintain emotional well-being, energy levels, ability to perform daily tasks and overall productivity.

## Results

### Sociodemographic characteristics of the study participants

A total of 214 study participants took part in this study; and the majority 153 (71.5%) of them were from TASH. Most 161 (75.5%) were female patients with a female-to-male ratio of 3 to 1. Regarding the age distribution, the median age of the study participants was 30 years and ranged from 15 to 88 years, and most 78 (36.4%) participants were in the 25–34 years age group. One-third of the study participants had a university degree or more 76 (35.5%), and half of them 109 (50.9%) lived far from the hematology clinic (outside Addis Ababa) (Table 1).

**Table 1:** Sociodemographic characteristics of ITP patients attending the TASH and SPHMMC hematology clinics in Addis Ababa, Ethiopia, 2022 (n = 214).

Variables	Frequency	Percentage
Study site		
TASH	153	71.5
SPHMMC	61	28.5
Sex		
Female	161	75.2
Male	53	24.8
Age		
14–24	58	27.1
25–34	78	36.5
35–44	40	18.7
45–54	20	9.3
55 and above	18	8.4
Educational level		
Unable to read and write	7	3.3
Enable read and write	12	5.6
Primary education (grades 1–8)	33	15.4
Secondary Education (grades 9–12)	54	25.2
Diploma/certificate	32	15.0
Degree and above	76	35.5
Residence		
Outside Addis Ababa	109	50.9
Addis Ababa	105	49.1

*TASH* Tikur Anbessa Specialized Hospital, *SPHMMC* St. Paul’s Hospital Millennium Medical College.

### Clinical characteristics of ITP patients during diagnosis

The clinical characteristics of the study participants are shown in Table 2. During the assessment, the most common symptom of ITP was fatigue 53 (25.2%), followed by headache 14 (6.5%) and depression 8 (3.7%). Common clinical symptoms during diagnosis include epistaxis and wet purpura (mucosal bleeding) 166 (77.6%), followed by fatigue 157 (73.4%) and skin manifestations (petechiae and ecchymosis) 120 (56.1%).

**Table 2:** Clinical characteristics of ITP patients attending the TASH and SPHMMC hematology clinics in Addis Ababa, Ethiopia, 2022 (n = 214).

Variables	Frequency	Percentage
Comorbidity		
Yes	91	42.5
No	123	57.5
Current symptoms of ITP		
Fatigue	54	25.2
Headache	14	6.5
Depression	8	3.7
Weight loss	3	1.4
Bleeding	1	0.5
Clinical presentations during diagnosis		
Epistaxis and wet purpura	166	77.6
Fatigue	157	73.4
Skin manifestation	120	56.1
Heavy menstrual bleeding	59	27.6
Signs of anemia (pallor)	54	25.2
Severe bleeding*	12	5.6

*Severe bleeding gastrointestinal bleeding, Intracranial bleeding, rectal bleeding, retinal hemorrhage.

**Figure 1:**
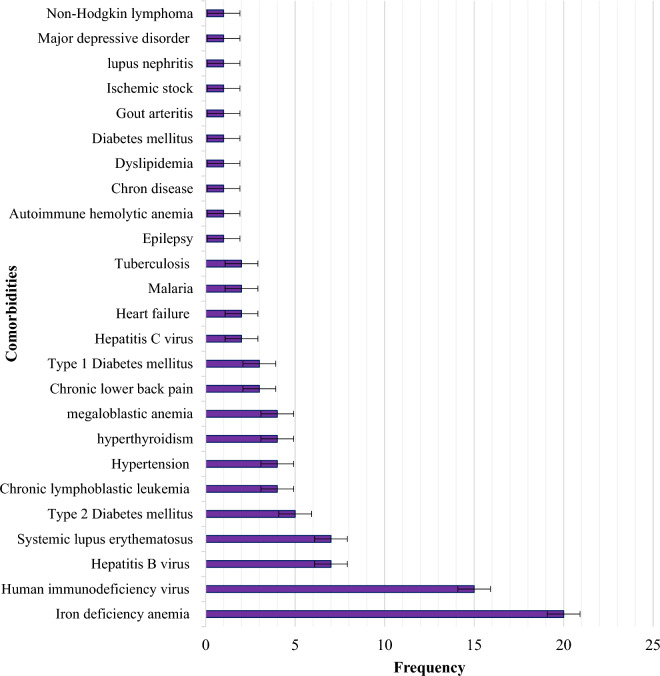
Comorbidities in ITP patients attending the TASH and SPHMMC hematology clinics in Addis Ababa, Ethiopia, 2022 (n = 214).

Of the total study participants, 91 (42.5%) patients had comorbidities and Iron deficiency anemia 20 (22.0%), followed by HIV 15 (16.5%), HBV 7 (7.7%), and Systemic Lupus Erythematous (SLE) 7 (7.7%) that accounted for the highest proportion of comorbidities in ITP patients attending TASH and SPHMMC during the study period (Fig. 1).

The median age of study participants at diagnosis of ITP was 27 years (IQR 22.0–37.0), whereas the median duration of ITP since diagnosis was 24 months (IQR 12.0–60.0), and among study participants who experienced a relapse of ITP, the median duration of relapse was 12 months (IQR 6.0–24.0). The median value of primary clinical/laboratory findings at diagnosis, such as platelet count, was 15 × 10^9^/L (IQR 8.0–24.3 × 10^9^/L), hemoglobin was 13 g/dL (IQR 9.8–14.6 g/dL), and white blood cell count was 7.1 × 10^9^/L (IQR 5.0–10.0 × 10^9^/L) (Table 3).

**Table 3:** The medians of clinical characteristics in ITP patients attending the TASH and SPHMMC hematology clinics in Addis Ababa, Ethiopia, 2022 (n = 214).

Variables	Median (IQR)
Age at diagnosis in a year	27 (22.0–37.0 years)
Duration since ITP diagnosis in a month	24 (12.0–60.0 months)
Time of ITP relapse in months	12 (6.0–24.0 months)
Primary clinical/laboratory findings during diagnosis	
Baseline platelet count (× 10^9^/L)	15 (8.0–24.3)
Baseline hemoglobin count (g/dl)	13 (9.8–14.6)
Baseline white blood cell count (× 10^9^/L)	7.1 (5.0–10.0)
Most recent platelet count (× 10^9^/L)	146 (59.5–202.5)

**Figure 2:**
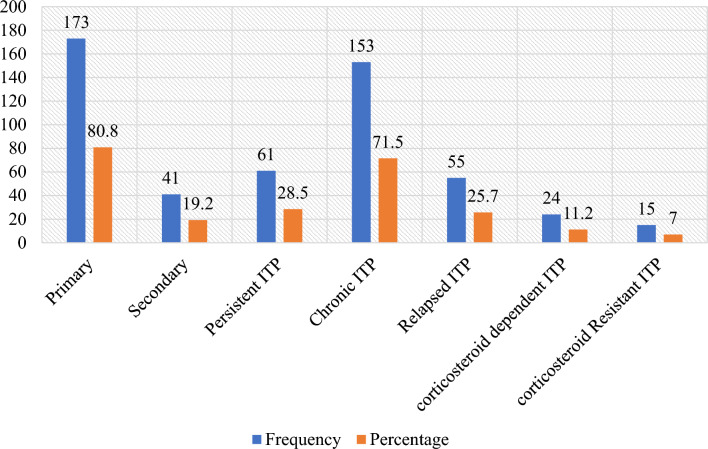
Classification of ITP according to the 2019 ASH guidelines of ITP patients attending the TASH and SPHMMC hematology clinics in Addis Ababa, Ethiopia, 2022 (n = 214).

The majority 173 (80.8) of study participants had primary ITP, and regarding the phase of ITP, two-thirds (n = 153) of study participants had chronic ITP. In addition, 24 (11.2%) and 15 (7.0%) of study participants had corticosteroid-dependent and corticosteroid-resistant ITP, respectively. After completing the first-line treatment of ITP, 55 (25.7%) patients relapsed within a median time of 12 months (Fig. 2).

### Secondary cause of ITP

**Figure 3:**
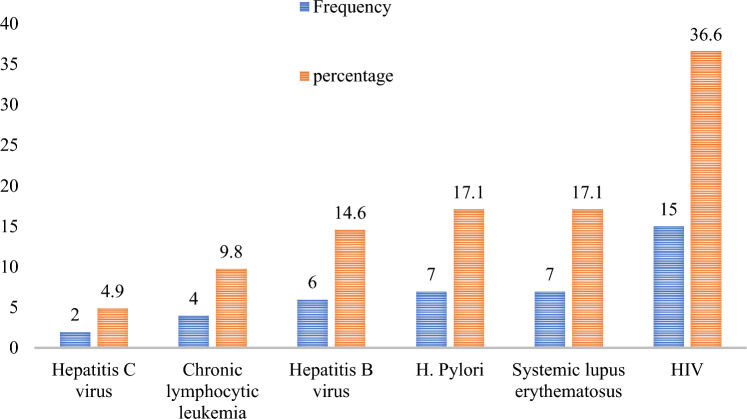
Secondary causes of ITP patients attending the TASH and SPHMMC hematology clinics in Addis Ababa, Ethiopia, 2022 (n = 214).

Of the total study participants, only 41 patients (19.2%) had secondary ITP and Human immunodeficiency virus (HIV) 15 (36.6%) accounted for the largest proportion of secondary causes of ITP, followed by systemic lupus erythematous (SLE) 7 (17.1%) and *H. pylori* infection 7 (17.1%) (Fig. 3).

### Treatment-related characteristics of ITP

For the treatment of ITP, the majority 172 (80.4%) of study participants took prednisolone alone as firstline treatement followed by combinations of prednisolone and dexamethasone 31 (14.5%). [Azathioprine or rituximab] + prednisolone 20 (36.4%) were the most commonly prescribed medications as second-line treatment options for ITP.

About 63 (29.4%) of the study participants received platelet transfusions to prevent bleeding, and 27 (12.6%) took tranexamic acid to stop bleeding. In addition, 121 (56.5%), 100 (46.7%), and 45 (21.0%) study participants took cotrimoxazole prophylaxis (CPT), proton pump inhibitors (PPI), and calcium with vitamin D3 supplement as prophylaxis to prevent immunosuppression-related infections, peptic ulcers, and osteoporosis, respectively (Table 4).

**Table 4:** Treatment-related characteristics of ITP patients attending the TASH and SPHMMC hematology clinics in Addis Ababa, Ethiopia, 2022 (n = 214).

Variables	Frequency	Percentage
First-line treatment of ITP		
Prednisolone alone	172	80.4
Prednisolone + Dexamethasone	31	14.5
Dexamethasone alone	6	2.8
Prednisolone + Methylprednisolone	5	2.3
Second-line treatments of ITP		
[Azathioprine or Rituximab] + Prednisolone	20	36.4
[Rituximab alone] or [Prednisolone alone] or [Azathioprine alone]	10	18.2
Rituximab + Splenectomy + [Azathioprine or Prednisolone]	8	14.5
[Rituximab + Azathioprine] ± Prednisolone	7	12.7
[Splenectomy + Prednisolone] or [Splenectomy + Rituximab]	6	10.9
[Splenectomy + Azathioprine + Prednisolone] ± Rituximab	4	7.3
Other medications to stop bleeding		
Platelet transfusion	63	29.4
Tranexamic acid	27	12.6
For prophylaxis of corticosteroid complications		
Cotrimoxazole prophylaxis treatment	121	56.5
Proton pump inhibitors	100	46.7
Calcium with Vitamin D3 supplementation	45	21.0

### Common corticosteroid side effects in ITP patients

Regarding corticosteroid side effects, 182 (85.1%) developed physical appearance related (Weight gain ⁄ increased appetite, moon face, bloating, swelling, stretch mark, acne and hair loss) corticosteroid side effects and 107 (50.0%) emotionally related (insomnia, restlessness, sleeping, depression, anxiety, anger and irritability) corticosteroid side effects. In addition, corticosteroid-related complications such as elevated blood glucose 19 (8.9%), elevated blood pressure 15 (7.0%), iatrogenic Cushing’s syndrome 9 (4.2%) and osteoporosis 5 (2.3%) occurred in the study participants (Table 5).

**Table 5:** Common corticosteroid side effects in ITP patients attending the TASH and SPHMMC hematology clinics in Addis Ababa, Ethiopia, 2022 (n = 214).

Corticosteroid side effects/complications	Frequency	Percentage
Physical appearance-related (weight gain/increased appetite, moon face, bloating, swelling, stretch mark, acne and hair loss)	182	85.1
Emotional-related (insomnia, restlessness, sleeping, depression, anxiety, anger and irritability)	107	50.0
Increase blood glucose	19	8.9
Increase blood pressure	15	7.0
Iatrogenic Cushing’s syndrome	9	4.2
Osteoporosis	5	2.3

### Medication adherence of ITP patients by using the Morisky Green Levine scale

**Figure 4:**
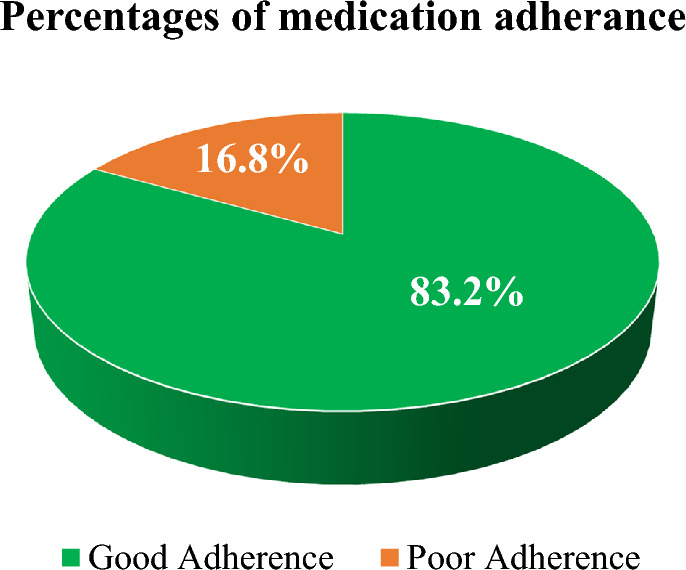
Medication adherence by using the Morisky Green Levine scale in ITP patients attending the TASH and SPHMMC hematology clinics in Addis Ababa, Ethiopia, 2022 (n = 214).

According to the Morisky Green Levine scale, 178 (83.2%) of the study participants had good adherence to their ITP medications (Fig. 4).

### Treatment response of ITP patients

Response to treatment was assessed at 3, 6, and 12 months after treatment initiation. Of the included participants assessed at a different time point, 139 (65.0%), 127 (69.8%), and 109 (76.2%) had complete responses at 3 months, 6 months, and 12 months, respectively (Table 6).

**Table 6:** Treatment response of ITP patients attending the TASH and SPHMMC hematology clinics in Addis Ababa, Ethiopia, 2022 (n = 214).

First line drugs	Treatment responses of ITP at 3 months (n = 214)			
	Complete response (CR), N (%)	Partial response (PR), N (%)	No response (NR), N (%)	Total N (%)
Prednisolone alone	117 (68.0%)	43 (25.0%)	12 (7.0%)	172
Prednisolone + Dexamethasone	15 (48.4%)	8 (25.8%)	8 (25.8%)	31
Prednisolone + methylprednisolone	4 (80.0%)	1 (20.0%)	0	5
Dexamethasone alone	3 (50.0%)	2 (33.3%)	1 (16.7%)	6
Total response at 3 months of treatment (n = 214)	139 (65.0%)	54 (25.2%)	21 (9.8%)	214
Treatment responses of ITP at 6 months (n = 182)				
Prednisolone alone	109 (72.7%)	33 (22.0%)	8 (5.3%)	150
Prednisolone + Dexamethasone	12 (50.0%)	8 (33.3%)	4 (16.7%)	24
Prednisolone + Methylprednisolone	4 (80.0%)	1 (20.0%)	0	5
Dexamethasone alone	2 (66.7%)	1 (33.3%)	0	3
Total response at 6 months of treatment (n = 182)	127 (69.8%)	43 (23.6%)	12 (6.6%)	182
Treatment responses of ITP at 12 months (n = 143)				
Prednisolone alone	91 (75.8%)	21 (17.5%)	8 (6.7%)	120
Prednisolone + Dexamethasone	13 (76.5%)	3 (17.6)	1 (5.9%)	17
Prednisolone + methylprednisolone	4 (80.0%)	0	1 (20.0%)	5
Dexamethasone alone	1 (100%)	0	0	1
Total responses at 12 months of treatment (n = 143)	109 (76.2%)	24 (16.8%)	10 (7.0%)	143

### Predicting factors for ITP treatment response

Fourteen variables (study site, sex, age, residence, comorbidities, baseline platelet count, Side effects of corticosteroid, heavy menstrual bleeding, H.pylori, HBV, HIV, SLE, signs of anemia at presentation, adherence, and HRQoL) were included in the univariate analysis. Of these six variables (study site, heavy menstrual bleeding, Signs of anemia at presentations, H.pylori, HBV and HRQoL) that met the inclusion criteria for multinomial logistic regression analysis, only four variables (study site, heavy menstrual bleeding, HBV and HRQoL) were significantly associated with ITP treatment response.

As the negative impact of ITP on health related quality of life (HRQoL) increased by one unit, the odds of having partial response were increased by 1.22 times (AOR = 1.221, 95% CI 1.096–1.360, *p* < 0001) compared to complete response. The odds ratio for partial response in relation to complete response in patients having heavy menstrual bleeding during diagnosis was 2.255 (AOR = 2.255, 95% CI 0.925–5.497, *p* = 0.025) times more likely compared to those not having heavy menstrual bleeding. ITP patients who visited TASH were 56.9% less likely to have a partial response (AOR = 0.431, 95% CI 0.197–0.941, *p* = 0.035) than patients who visited SPHMMC compared to a complete response. Furthermore, Hepatitius B virus (HBV)-negative ITP patients were 94.8% less likely to have no response (AOR = 0.052, 95% CI 0.004–0.621, *p* = 0.02) than HBV-postive ITP patients compared to complete response. The model containing the full set of predictors represents a significant improvement in fit relative to a null model (model *x*^2^ = 228.505, *p* < 0.001). In addition, Pearson and deviance statistics were also much higher, with *p* values of (*p* = 0.98) and (*p* = 1.0), respectively, which means that the model is a good fit for the data. In addition, overdispersion is not a problem for the model since *p* = 0.98 and *p* = 1.0 are much higher than 0.05 (Table 7).

**Table 7:** Predictive factors for treatment response of ITP patients attending the TASH and SPHMMC hematology clinics in Addis Ababa, Ethiopia, 2022 (n = 214).

Variables	Category	B (SE)	COR of 95% CI	B (SE)	AOR of 95% CI	*p* value
Partial response versus complete response						
HRQoL		0.022 (0.018)	1.022 (0.988–1.058)	0.200 (0.055)	1.221 (1.096–1.360)	0.000*
Study site	SPHMMC		1		1	
	TASH	− 0.411 (0.339)	0.663 (0.341–1.289)	− 0.842 (0.399)	0.431 (0.197–0.941)	0.035*
Heavy menstrual bleeding	No		1		1	
	Yes	0.540 (0.398)	1.716 (0.787–3.743)	0.813 (0.455)	2.255 (0.925–5.497)	0.025*
*Helicobacter pylori*	Negative		1		1	
	Positive	− 0.556 (0.927)	0.574 (0.093–3.531)	− 0.582 (1.148)	0.559 (0.059–5.298)	0.612
HBV	Negative		1		1	
	Positive	0.451 (1.130)	1.570 (0.172–14.376)	− 0.418 (1.191)	0.658 (0.064–6.790)	0.725
Signs of anemia at presentations	No		1		1	
	Yes	− 0.335 (0.367)	0.716 (0.348–1.470)	− 0.601 (0.435)	0.548 (0.234–1.286)	0.167
No response versus complete response						
HBV	Negative		1		1	
	Positive	− 1.727 (0.804)	0.178 (0.037–0.859)	− 2.957 (1.266)	0.052 (0.004–0.621)	0.02*
HRQoL		0.075 (0.025)	1.078 (1.027–1.131)	0.117 (0.067)	1.125 (0.985–1.283)	0.081
Study site	SPHMMC		1		1	
	TASH	− 2.970 (0.725)	3.705 (0.824–16.659)	1.969 (1.044)	7.166 (0.927–55.40)	0.059
Heavy menstrual bleeding	No		1		1	
	Yes	− 0.846 (0.476)	0.429 (0.169–1.090)	0.021 (0.664)	1.021 (0.278–3.750)	0.975
Signs of anemia at presentations	No		1		1	
	Yes	− 1.002 (0.487)	0.367 (0.141–0.953)	− 0.703 (0.673)	0.495 (0.132–1.853)	0.297
*Helicobacter pylori*	Negative		1		1	
	Positive	− 2.022 (0.854)	0.132 (0.025–0.706)	− 2.106 (1.192)	0.122 (0.012–1.259)	0.077

R^2^ = 0.694 (Cox and Snell), 0.847 (Nagelkerke), 0.691 (McFadden), Model x^2^ = 228.505, Pearson (*p* = 0.98) and deviance (*p* = 1.0), *p < 0.05.

*SE* Standard error, *HRQoL* Health-related quality of life, *TASH* Tikur Anbessa Specialized Hospital, *SHMMC* St. Paulo’s Hospital Millennium Medical College, *HBV* Hepatitis B virus.

## Discussion

The present study aimed to investigate the clinical outcomes of different ITP treatment regimens in patients with ITP and factors related to treatment outcomes. The updated international consensus report indicates that there are differences in ITP clinical presentations, clinical outcomes, and treatment responses^32^. Therefore, the clinical outcomes and complications of corticosteroid treatment were evaluated. In addition, steroids are currently the standard first-line treatment for adults diagnosed with ITP^9^. Steroids are also associated with numerous bothersome side effects that may cause patients to discontinue treatment by themselves or poorly adherent and the most common reason for healthcare professional led treatment modification(discontinue, reduce dose or change of treatment)^33^.

Most epidemiologic data suggest that women are more commonly affected by ITP during their childbearing age and that the prevalence after menopause is similar to that of men^6–8,43,44^. In this study, 75.5% of the study participants were female, which is consistent with other studies from the United States of America (76%)^45^, Turkey (71.3%)^46^, and Malaysia (71.8%)^36^. In our study, the proportion of females was higher than that in studies conducted in the United Kingdom (56.9%)^8^, Germany (57%)^47^, China (63.6%)^48^, and in the ITP World Impact Survey data (65%)^17^ but lower than that in studies conducted in Mexico (81.8%)^49^ and Egypt (84%)^50^. The most common clinical presentation at diagnosis of ITP was epistaxis and wet purpura (mucosal bleeding) (77.6%), followed by fatigue (73.4%), skin manifestations (petechiae and ecchymosis) (56.1%), and heavy menstrual bleeding (27.6%). This was similar to studies conducted in Mexico^39^, Spain^20^, France[21)], Turkey^46^ and the ITP impact world survey^17^.

In this study the median age of ITP patients during diagnosis of ITP was 27 years (IQR 22.0–37.0); this was lower than studies conducted in Twain at 45.5 years^51^, in the United States of America at 50 years^22^, in Spain at 58 years^37^, and in Germany at 55 years^47^. In addition the median time to relapse after starting initial treatment for ITP was 12 months (IQR 6.0–24.0), which was higher than studies conducted in Taiwan 9.5 months^51^ and Mexico 2 months^52^. On the other hand, the median time to relapse was lower than in the Norway study, where the median relapse was 17 months^53^.

In this study, 19.2% of study participants had secondary ITP, which is comparable with studies conducted in Malaysia (23%)^36^ and higher than studies conducted in Germany (9%)^47^. From the causes of secondary ITP, HIV (36.6%), followed by SLE (17.1%) and *H. pylori* infection (17.1%), accounted for the highest number of underlying disease conditions. On the other hand, studies conducted in Spain^20^; SLE (17.6%) followed by lymphoproliferative syndromes (17.6%); in Mexico^52^, SLE (34.8%), infection (26.1%), and thyroid disease (17.3%); in Taiwan^51^; Evans’ syndrome (33.3%), hepatitis C virus (28.6%) and SLE 28.6%; and in Malaysia^36^, autoimmune disease (15.8%) and viral infections (4.4%) were the most common underlying diseases.

Corticosteroids remain the most commonly used first-line treatment, followed by IVIg for the management of ITP^9^. In this study, all ITP patients (100%) received corticosteroids, either prednisolone alone or dexamethasone and methylprednisolone, followed by prednisolone because IVIg was not available at TASH and SPHMMC. A similar study was conducted in Malaysia, where 98.8% of the study participants were taking steroids and the remaining 1.2% were taking IVIg^36^. On the other hand, in a study conducted in China, 78.5% of ITP patients received corticosteroids, and 8.0% of ITP patients received IVIg as first-line treatment^54^. Two studies conducted in Spain showed that 40.6% and 64.3% of ITP patients also received corticosteroid monotherapy^20,37^. In a study conducted in Mexico, only 33.3% were treated with steroids alone; the remaining 28.4% received low-dose rituximab plus steroids, 13.8% danazol plus steroids, and 8.9% eltrombopag plus corticosteroids^39^.

In this study, the a complete response rate to treatment at three months of treatment initiation was (65.0%) whcih was lower as compared with the study conducted in Iran (80.0%)^55^. This may be because, in the Iranian study, all participants received high-dose dexamethasone, which is more effective than conventional corticosteroid therapy as initial treatment in newly diagnosed ITP and has fewer relapses and toxicities^56^. On the other hand, the response rate was higher than that in a study conducted in Malaysia (36.5%)^36^. In this study, a complete treatment response rates at 6 months after treatment initiation was also (69.8%) which was lower than studies conducted in Iran (73.3%)^55^ and spain (76.2%)^20^. On the other hand, this study has a higher complete response rate than the study conducted in Malaysia (26.1%)^36^. This may be because in Malaysia, 169 (41.9%) of the study participants were lost to follow-up at 12 months treatment initiation.

In this study, the multinomial logistic regression analysis indicated that having heavy menstrual bleeding during diagnosis and reduced HRQoL were association with partial response and this might be due to 52 (88.2%) of women who reported heavy menstrual bleeding had a platelet count below 30 × 109/L, which would typically require corticosteroids + IVIg treatment to increase platelet levels rapidly. However, IVIg treatment was not available in the study setting. Patients with reduced HRQoL tend to have a partial response to treatment and may negatively impact the overall treatment outcomes of patients with ITP. Improving the platelet count can lead to an improved HRQoL by reducing patients' concerns and fears of bleeding, minimizing fatigue, and increasing their ability to participate in activities. According to this study, patients who had visited TASH were found to be 56.9% less likely to have a partial response compared to patients who visited SPHMMC. This could be attributed to the absence of a national treatment guideline for teaching hospitals in Ethiopia, leading to variations in treatments across various institutions. In this study, patients who tested positive for HBV 94.8% had no response. In patients with ITP, HBV exposure was found to be associated with greater disease severity and hospitalization rates^57^, and may be considered a predictor for poor response to ITP-specific treatments.

This study evaluated the treatment outcomes of ITP and adherence to treatment in the Ethiopian population. In addition, the study assessed the complications of corticosteroid side effects in ITP patients. Finally, this study had certain limitations. since the sample size was small and consequetive sampling technique was used, it may affect the generalizability of the finding to patients treated elsewhere. In addition, the report on treatment adherence is based on the information provided by the patients and other factors that may influence treatment adherence were not addressed.

## Conclusion

The highest complete response rate was achieved at 12 months compared to at 3 and 6 months, and they adhered to their treatments. The predictive factors for the partial response of ITP patients at 3 months were HRQoL, study site and heavy menstrual bleeding. HBV-positive ITP patients were also predictive factors for no response of ITP patients. The side effects of corticosteroids also affect the response to treatment of ITP patients. In general, concerted efforts must be made to reduce/manage corticosteroid related side effects.

## Data Availability

The datasets used during the current study are available from the corresponding author upon reasonable request.

## Abbreviations

AOR: Adjusted odds ratio
ASH: American Society of Hematology
CI: Confidence interval
CLL: Chronic lymphocytic leukemia
COR: Crude odds ratio
COVID-19: Coronavirus disease
CPT: Cotrimoxazole prophylaxis
CR: Complete response
*H. pylori*: *Helicobacter pylori*
HBV: Hepatitis B virus
HIV: Human immunodeficiency virus
HMIS: Health Management Information System
HRQoL: Health related quality of life
ITP: Immune thrombocytopenia
IVIg: Intravenous immunoglobulin
LS: Laparoscopic splenectomy
PPI: Proton pump inhibitors
PR: Partial response
MGL: Morisky Green Levine scale
NR: No response
SPHMMC: St Poulos Hospital Millennium Medical College
TASH: Tikur Anbessa Specialized Hospital
TPO-RAs: Thrombopoietin-receptor agonists
SLE: Systemic lupus erythematous

## References

[1] Marini I, Zlamal J, Pelzel L, Bethge W, Faul C, Holzer U (2019). Autoantibody mediated desialylation impairs human thrombopoiesis and platelet life span. Blood..

[2] Rodeghiero F, Stasi R, Gernsheimer T, Michel M, Provan D, Arnold DM (2009). Standardization of terminology, definitions and outcome criteria in immune thrombocytopenic purpura of adults and children: Report from an international working group. Blood J. Am. Soc. Hematol..

[3] Ozelo MC, Colella MP, de Paula EV, do Nascimento ACKV, Villaça PR, Bernardo WM (2018). Guideline on immune thrombocytopenia in adults: Associação Brasileira de hematologia, hemoterapia e terapia cellular. Project guidelines: Associação médica Brasileira–2018. Hematol. Transfus. Cell Therapy.

[4] Lev, P. R., Goette, N. P. & Marta, R. F. Pathophysiological mechanisms leading to low platelet count in immune thrombocytopenia (2020).

[5] Cines DB, McMillan R (2007). Pathogenesis of chronic immune thrombocytopenic purpura. Curr. Opin. Hematol..

[6] Andrès E, Mecili M, Fothergill H, Zimmer J, Vogel T, Maloisel F (2012). Gender-related analysis of the clinical presentation, treatment response and outcome in patients with immune thrombocytopenia. La Presse Medicale..

[7] Gernsheimer T (2008). Epidemiology and pathophysiology of immune thrombocytopenic purpura. Eur. J. Haematol..

[8] Marieke Schoonen W, Kucera G, Coalson J, Li L, Rutstein M, Mowat F (2009). Epidemiology of immune thrombocytopenic purpura in the General Practice Research Database. Br. J. Haematol..

[9] Neunert C, Terrell DR, Arnold DM, Buchanan G, Cines DB, Cooper N (2019). American Society of Hematology 2019 guidelines for immune thrombocytopenia. Blood Adv..

[10] Chiao EY, Engels EA, Kramer JR, Pietz K, Henderson L, Giordano TP (2009). Risk of immune thrombocytopenic purpura and autoimmune hemolytic anemia among 120 908 US veterans with hepatitis C virus infection. Arch. Intern. Med..

[11] Cines DB, Bussel JB, Liebman HA, Luning Prak ET (2009). The ITP syndrome: Pathogenic and clinical diversity. Blood J. Am. Soc. Hematol..

[12] Mitta A, Curtis BR, Reese JA, George JN (2019). Drug-induced thrombocytopenia: 2019 update of clinical and laboratory data. Am. J. Hematol..

[13] Rajan SK, Espina BM, Liebman HA (2005). Hepatitis C virus-related thrombocytopenia: Clinical and laboratory characteristics compared with chronic immune thrombocytopenic purpura. Br. J. Haematol..

[14] Shah I (2013). Immune thrombocytopenic purpura: A presentation of HIV infection. J. Int. Assoc. Provid. AIDS Care (JIAPAC).

[15] Kewan T, Gunaratne TN, Mushtaq K, Alayan D, Daw H, Haddad A (2021). Outcomes and management of immune thrombocytopenia secondary to COVID-19: Cleveland clinic experience. Transfusion..

[16] Farid J, Gul N, Idris M (2012). Clinical presentations in immune thrombocytopenic purpura. J. Ayub Med. Coll. Abbottabad.

[17] Cooper N, Kruse A, Kruse C, Watson S, Morgan M, Provan D (2021). Immune thrombocytopenia (ITP) World Impact Survey (iWISh): Patient and physician perceptions of diagnosis, signs and symptoms, and treatment. Am. J. Hematol..

[18] Zitek T, Weber L, Pinzon D, Warren N (2022). Assessment and management of immune thrombocytopenia (ITP) in the emergency department: Current perspectives. Open Access Emerg. Med..

[19] Jaime-Pérez JC, Aguilar-Calderón P, Jiménez-Castillo RA, Ramos-Dávila EM, Salazar-Cavazos L, Gómez-Almaguer D (2020). Treatment outcomes and chronicity predictors for primary immune thrombocytopenia: 10-year data from an academic center. Ann. Hematol..

[20] Palau J, Sancho E, Herrera M, Sánchez S, Mingot ME, Upegui RI (2017). Characteristics and management of primary and other immune thrombocytopenias: Spanish registry study. Hematology..

[21] Grimaldi-Bensouda L, Nordon C, Michel M, Viallard J-F, Adoue D, Magy-Bertrand N (2016). Immune thrombocytopenia in adults: a prospective cohort study of clinical features and predictors of outcome. Haematologica..

[22] Newton JL, Reese JA, Watson SI, Vesely SK, Bolton-Maggs PH, George JN (2011). Fatigue in adult patients with primary immune thrombocytopenia. Eur. J. Haematol..

[23] Terrell DR, Neunert CE, Cooper N, Heitink-Pollé KM, Kruse C, Imbach P (2020). Immune thrombocytopenia (ITP): Current limitations in patient management. Medicina..

[24] Kruse C, Kruse A, Watson S, Morgan M, Cooper N, Ghanima W (2018). Patients with immune thrombocytopenia (ITP) frequently experience severe fatigue but is it under-recognized by physicians: Results from the ITP World Impact Survey (I-WISh). Blood..

[25] Kwiatkowska A, Radkowiak D, Wysocki M, Torbicz G, Gajewska N, Lasek A (2019). Prognostic factors for immune thrombocytopenic purpura remission after laparoscopic splenectomy: A Cohort study. Medicina..

[26] Chugh S, Darvish-Kazem S, Lim W, Crowther MA, Ghanima W, Wang G (2015). Rituximab plus standard of care for treatment of primary immune thrombocytopenia: A systematic review and meta-analysis. Lancet Haematol..

[27] Wang J, Li Y, Wang C, Zhang Y, Gao C, Lang H, et al. Efficacy and safety of the combination treatment of rituximab and dexamethasone for adults with primary immune thrombocytopenia (ITP): A meta-analysis. BioMed Res. Int. **2018** (2018). 10.1155/2018/1316096 PMC6311778 30648105

[28] Trotter P, Hill QA (2018). Immune thrombocytopenia: Improving quality of life and patient outcomes. Patient Relat. Outcome Meas..

[29] Grainger JD, Young NL, Blanchette VS, Klaassen RJ (2013). Quality of life in immune thrombocytopenia following treatment. Arch. Dis. Child..

[30] Vecchio, R. & Intagliata, E. Idiopathic thrombocytopenic purpura: Current therapeutical strategies and review of the literature on outcome after splenectomy. Ann. Laparosc. Endosc. Surg. 2020;7.

[31] Kashiwagi H, Tomiyama Y (2013). Pathophysiology and management of primary immune thrombocytopenia. Int. J. Hematol..

[32] Provan D, Arnold DM, Bussel JB, Chong BH, Cooper N, Gernsheimer T (2019). Updated international consensus report on the investigation and management of primary immune thrombocytopenia. Blood Adv..

[33] Brown TM, Horblyuk RV, Grotzinger KM, Matzdorff AC, Pashos CL (2012). Patient-reported treatment burden of chronic immune thrombocytopenia therapies. BMC Blood Disord..

[34] Provan D, Newland AC (2015). Current management of primary immune thrombocytopenia. Adv. Therapy.

[35] Qu M, Liu Q, Zhao H-G, Peng J, Ni H, Hou M (2018). Low platelet count as risk factor for infections in patients with primary immune thrombocytopenia: A retrospective evaluation. Ann. Hematol..

[36] Hamzah R, Yusof N, Tumian NR, Abdul Aziz S, Mohammad Basri NS, Leong TS (2022). Clinical epidemiology, treatment outcome and mortality rate of newly diagnosed immune thrombocytopenia in adult multicentre study in Malaysia. J. Blood Med..

[37] Lozano ML, Revilla N, Gonzalez-Lopez T, Novelli S, González-Porras J, Sanchez-Gonzalez B (2016). Real-life management of primary immune thrombocytopenia (ITP) in adult patients and adherence to practice guidelines. Ann. Hematol..

[38] Mahévas M, Gerfaud-Valentin M, Moulis G, Terriou L, Audia S, Guenin S (2016). Characteristics, outcome, and response to therapy of multirefractory chronic immune thrombocytopenia. Blood J. Am. Soc. Hematol..

[39] Jaime-Pérez JC, Aguilar-Calderón P, Jiménez-Castillo RA, Ramos-Dávila EM, Salazar-Cavazos L, Gómez-Almaguer D (2020). Treatment outcomes and chronicity predictors for primary immune thrombocytopenia: 10-year data from an academic center. Ann. Hematol..

[40] Morisky DE, Green LW, Levine DM (1986). Concurrent and predictive validity of a self-reported measure of medication adherence. Med. Care..

[41] Culig J, Leppee M (2014). From Morisky to Hill-bone; Self-reports scales for measuring adherence to medication. Coll. Antropol..

[42] Lam, W. Y. & Fresco, P. Medication adherence measures: An overview. BioMed Res. Int. **2015** (2015). 10.1155/2015/217047 PMC4619779 26539470

[43] Kurata Y, Fujimura K, Kuwana M, Tomiyama Y, Murata M (2011). Epidemiology of primary immune thrombocytopenia in children and adults in Japan: A population-based study and literature review. Int. J. Hematol..

[44] Michel M (2009). Immune thrombocytopenic purpura: Epidemiology and implications for patients. Eur. J. Haematol..

[45] Snyder CF, Mathias SD, Cella D, Isitt JJ, Wu AW, Young J (2008). Health-related quality of life of immune thrombocytopenic purpura patients: Results from a web-based survey. Curr. Med. Res. Opin..

[46] Pamuk G, Pamuk Ö, Başlar Z, Öngören Ş, Soysal T, Ferhanoğlu B (2002). Overview of 321 patients with idiopathic thrombocytopenic purpura: Retrospective analysis of the clinical features and response to therapy. Ann. Hematol..

[47] Weide R, Feiten S, Friesenhahn V, Heymanns J, Kleboth K, Thomalla J (2016). Outpatient management of patients with immune thrombocytopenia (ITP) by hematologists 1995–2014. Oncol. Res. Treat..

[48] Zhou Z, Yang L, Chen Z, Chen X, Guo Y, Wang X (2007). Health-related quality of life measured by the Short Form 36 in immune thrombocytopenic purpura: A cross-sectional survey in China. Eur. J. Haematol..

[49] Gómez-Almaguer D, Tarín-Arzaga L, Moreno-Jaime B, Jaime-Pérez JC, Ceballos-López AA, Ruiz-Argüelles GJ (2013). High response rate to low-dose rituximab plus high-dose dexamethasone as frontline therapy in adult patients with primary immune thrombocytopenia. Eur. J. Haematol..

[50] Omar IM, Abuelela S, Emam N (2018). Value of pre-and post-treatment platelet indices in patients with immune thrombocytopenic purpura. J. Biosci. Med..

[51] Chang H, Tang TC, Hung YS, Li PL, Kuo MC, Wu JH (2018). Immune thrombocytopenia: Effectiveness of frontline steroids and comparison of azathioprine, splenectomy, and rituximab as second-line treatment. Eur. J. Haematol..

[52] Jaime-Pérez JC, Ramos-Dávila EM, Aguilar-Calderón P, Jiménez-Castillo RA, Gómez-Almaguer D (2021). Diagnoses, outcomes, and chronicity predictors of patients with secondary immune thrombocytopenia: Ten-year data from a hematology referral center. Revista de Investigación Clínica..

[53] Tjønnfjord E, Holme PA, Darne B, Khelif A, Waage A, Michel M (2020). Long-term outcomes of patients treated with rituximab as second-line treatment for adult immune thrombocytopenia–Follow-up of the RITP study. Br. J. Haematol..

[54] Wang L, Xu L, Hao H, Jansen AG, Liu G, Li H (2020). First line treatment of adult patients with primary immune thrombocytopenia: A real-world study. Platelets..

[55] Mashhadi MA, Kaykhaei MA, Sepehri Z, Miri-Moghaddam E (2012). Single course of high dose dexamethasone is more effective than conventional prednisolone therapy in the treatment of primary newly diagnosed immune thrombocytopenia. DARU J. Pharm. Sci..

[56] Mashhadi MA, Kaykhaei MA, Sepehri Z, Miri-Moghaddam E (2012). Single course of high dose dexamethasone is more effective than conventional prednisolone therapy in the treatment of primary newly diagnosed immune thrombocytopenia. DARU J. Pharm. Sci..

[57] Wang L, Li L, Li C, Hou Y, Xu M, Yu Y (2022). Significance of anti-HBc serological status in primary immune thrombocytopenia. Br. J. Haematol..

